# Quantifying Heteromer Partitioning Reveals Inflammation‐Dependent Redistribution of Microglial Adenosine A_2A_ and Cannabinoid CB_2_ Receptors

**DOI:** 10.1002/glia.70200

**Published:** 2026-07-21

**Authors:** Rafael Franco, Jaume Lillo, Christian Griñán‐Ferré, Mercè Pallàs, Rafael Rivas‐Santisteban

**Affiliations:** ^1^ CIBERNED (Center for Networked Biomedical Research on Neurodegenerative Diseases), Spanish National Institute of Health Carlos III Madrid Spain; ^2^ Department of Biochemistry and Molecular Biomedicine University of Barcelona Barcelona Spain; ^3^ Institute of Theoretical and Computational Chemistry (IQTCUB), School of Chemistry, University of Barcelona Barcelona Spain; ^4^ Department of Biochemistry and Physiology University of Barcelona Barcelona Spain; ^5^ Department of Pharmacology and Therapeutic Chemistry Institute of Neurosciences–University of Barcelona Barcelona Spain

**Keywords:** adenosine A_2A_ receptor, cannabinoid CB_2_ receptor, GPCR heteromerization, in situ proximity ligation, microglial activation, MolBoolean, neuroinflammation, precision pharmacology, receptor partitioning

## Abstract

G protein–coupled receptor (GPCR) heteromerization represents a key organizational mechanism in cell signaling, but it remains difficult to determine, in native cells, how receptor‐associated signals are distributed between non‐interacting and heteromer‐associated states. Here, we address this limitation by combining proximity ligation assay (PLA) with the newly applied MolBoolean methodology, enabling in situ quantification of the partitioning of adenosine A_2A_ and cannabinoid CB_2_ receptor‐associated signals between non‐interacting fractions and A_2A_–CB_2_ heteromeric complexes in primary microglia. We show that resting microglia contain detectable A_2A_–CB_2_ heteromers together with a substantial non‐interacting A_2A_‐associated signal fraction. Selective activation of either receptor promotes redistribution of the detectable receptor‐associated signal toward the heteromer‐associated fraction. Ligand‐induced redistribution also occurred in HEK‐293T cells expressing the two receptors. In contrast, pro‐inflammatory activation of primary microglia with LPS/IFN‐γ markedly changes the basal organization of the receptor system, increasing the proportion of MolBoolean‐detectable signal associated with A_2A_–CB_2_ complexes, with approximately 70% of the detectable receptor‐associated signal corresponding to heteromeric complexes. In this inflammatory context, further agonist‐induced repartitioning is strongly limited compared with that observed in resting microglia. These findings identify inflammation‐dependent receptor partitioning as a quantitatively measurable feature of microglial A_2A_ and CB_2_ receptor organization and provide a framework for interpreting how receptor context may influence future studies of A_2A_–CB_2_ pharmacology under neuroinflammatory conditions.

## Graphical abstract

Proinflammatory activation reorganizes microglial A_2A_ and CB_2_ receptors, promoting their incorporation into stable heteromers and blocking agonist‐induced redistribution. The results reveal those heteromers as potential therapeutic targets in inflammatory contexts.

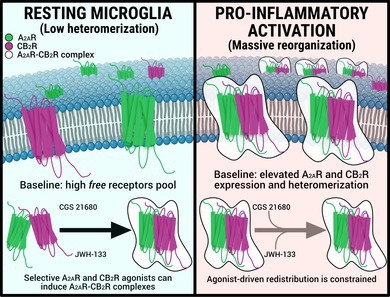

## 1 Introduction

G protein–coupled receptors (GPCRs) do not function exclusively as isolated monomers; many assemble into higher‐order complexes, including heteromers, thereby generating emergent pharmacology and signaling properties that cannot be inferred from either protomer alone. In the central nervous system, particularly in microglia, this organizational layer is likely to have important functional consequences, because purinergic and endocannabinoid pathways jointly regulate inflammatory tone, surveillance and motility programs, and the balance between neuroprotective and neurotoxic outcomes (Nakata et al. 2004; Prinster et al. 2005; Farran 2017; Guidolin et al. 2018; Navarro et al. 2018).

Among class A GPCRs, the adenosine A_2A_ receptor (A_2A_R) is notable for its broad heteromerization capacity. Several A_2A_R‐containing complexes have been described and pharmacologically characterized, including heteromers with the dopamine D_2_ receptor (Hillion et al. 2002; Fuxe et al. 2003; Bonaventura et al. 2015), the metabotropic glutamate mGlu5 receptor (Cabello et al. 2009), and the cannabinoid CB_1_ receptor (Carriba et al. 2007; Chiodi et al. 2016; Gonçalves‐Ribeiro et al. 2024). These complexes are particularly relevant to basal ganglia circuitry and, more broadly, to mechanisms controlling neuroinflammatory processes.

The heteromeric interaction between A_2A_R and the cannabinoid CB_2_ receptor (CB_2_R) is of particular interest. A_2A_R is closely associated with neuroinflammatory processes, can shape microglial reactivity, and is upregulated in microglia surrounding amyloid plaques in patients with Alzheimer's disease (Angulo et al. 2003; Saura et al. 2005; Madeira et al. 2015). CB_2_R, in turn, is widely viewed as a modulatory and potentially protective receptor enriched in activated microglia and has been pursued as a therapeutic target in neurodegeneration (de Lago and Fernández‐Ruiz 2007; Merighi et al. 2012; Wu et al. 2013; Young and Denovan‐Wright 2022). Consistent with this biological convergence, A_2A_R and CB_2_R have been reported to interact functionally and physically in microglia and other cellular contexts, suggesting that heteromerization may allow adenosinergic tone to gate cannabinoid signaling and vice versa (Franco et al. 2019).

A key limitation in the receptor‐heteromer field is quantitative and relates to interaction dynamics. When two GPCRs, A and B, can interact, there has been no practical way to determine, within the same biological sample, what fraction of A remains non‐interacting, or free, versus engaged in AB heteromers, and what fraction of B remains free versus engaged in AB heteromers. This uncertainty is not merely technical; it constrains biological and translational interpretation. A receptor that is predominantly free may differ in signaling, trafficking, and drug responsiveness from the same receptor when incorporated into a heteromeric complex. Thus, targeting a largely non‐interacting receptor pool may be fundamentally different from targeting a receptor population in which heteromeric complexes predominate, particularly in disease contexts.

Here, we address this quantitative gap in a biologically relevant system by examining how inflammatory activation changes the distribution of A_2A_R and CB_2_R between non‐interacting and heteromer‐engaged states in microglia. To this end, we combined proximity ligation assay (PLA), which reports A_2A_R–CB_2_R receptor proximity, with MolBoolean analysis, which enables simultaneous in situ quantification of non‐interacting A_2A_R, non‐interacting CB_2_R, and A_2A_R–CB_2_R heteromeric complexes within the same preparation. We first used HEK‐293T cells co‐expressing both receptors to establish whether selective receptor activation modifies A_2A_R–CB_2_R proximity and receptor partitioning. We then applied the same experimental logic to primary microglia, comparing resting cells with cells activated by lipopolysaccharide (LPS) plus interferon‐γ. This approach allowed us to determine whether pro‐inflammatory activation is associated primarily with changes in MolBoolean‐detectable receptor abundance or with redistribution of receptors between non‐ interacting and heteromer‐engaged states, and to assess how microglial activation modifies the capacity of acute receptor stimulation to further reshape receptor partitioning.

## 2 Results

### 2.1 Agonist Exposure Increases Detectable A_2A_ –CB_2_ Receptor Complexes in Heterologous Cells

**FIGURE 1:**
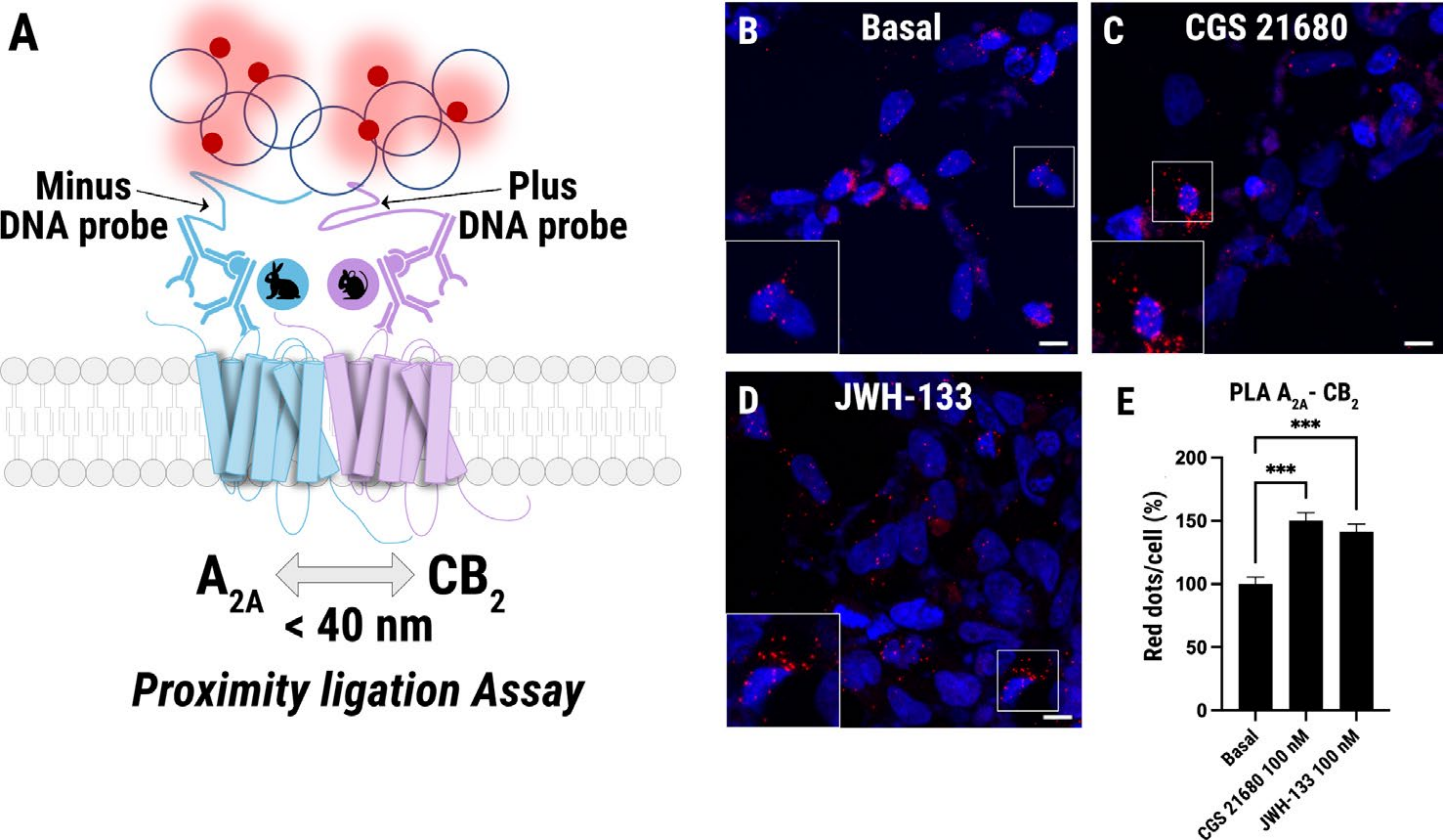
Selective agonist treatment promotes A_2A_–CB_2_ heteromerization in a heterologous system. Proximity Ligation Assays (PLA) were performed in HEK‐293T cells co‐expressing A_2A_ and CB_2_ receptors to detect and quantify heteromeric complexes. (A) Schematic representation of the PLA principle, showing detection of A_2A_ and CB_2_ receptors using species‐specific primary antibodies and corresponding DNA probes, which generate a fluorescent signal (red dots) upon ligation and amplification. Representative confocal microscopy images of PLA assays in HEK‐293T cells under basal conditions (B), or after stimulation (for 1 h) with the selective A_2A_R agonist, CGS 21680 (100 nM) (C) or the selective CB_2_ agonist, JWH‐133 (100 nM) (D). Red dots indicate A_2A_–CB_2_ heteromers; nuclei were stained with Hoechst (blue). Insets show zoomed regions. Scale bar = 15 μm. (E) Quantification of PLA signals. Data are expressed as the number of PLA‐positive red dots per cell, normalized to the basal condition, which was set to 100%. Bars represent mean ± SEM of single‐cell values pooled from *n* = 3 independent experiments. Statistical analysis was performed by one‐way ANOVA followed by Dunnett's post hoc test versus basal. The ANOVA showed a significant effect of treatment (*F*(2, 6) = 87.21; global *p* < 0.001), and Dunnett's test indicated significant differences versus basal (***p < 0.001).

To establish whether receptor activation dynamically regulates A_2A_–CB_2_ heteromer formation, we performed in situ proximity ligation assays (PLA) in HEK‐293T cells co‐expressing both receptors (Figure 1A). No detectable signal was observed in negative controls in which one primary antibody or the PLA probe was omitted (Supplementary Figure S1). Under basal conditions, constitutive A_2A_–CB_2_ heteromers were detected as discrete red fluorescent puncta (Figure 1B). Stimulation for 1 h with either the selective A_2A_R agonist CGS 21680 (100 nM; Figure 1C) or the selective CB_2_R agonist JWH‐133 (100 nM; Figure 1D) induced a robust increase in the density of PLA puncta per cell relative to basal conditions. These findings indicate that activation of either protomer promotes the assembly and/or stabilization of the heteromeric complex (Figure 1E). However, although PLA demonstrates an increase in A_2A_–CB_2_ receptor complexes, it does not detect non‐interacting receptor populations. Therefore, the precise proportion of *free* versus heteromer‐forming A_2A_ and CB_2_ receptors remains to be determined. The observed increase in red puncta per cell may reflect the recruitment or redistribution of previously non‐interacting receptors into heteromeric complexes.

### 2.2 MolBoolean Resolves A_2A_ ^free^, CB_2_ ^free^, and A_2A_ –CB_2_ ^het^ Signal Fractions in HEK‐293T Cells and Reveals Agonist‐Driven Repartitioning

**FIGURE 2:**
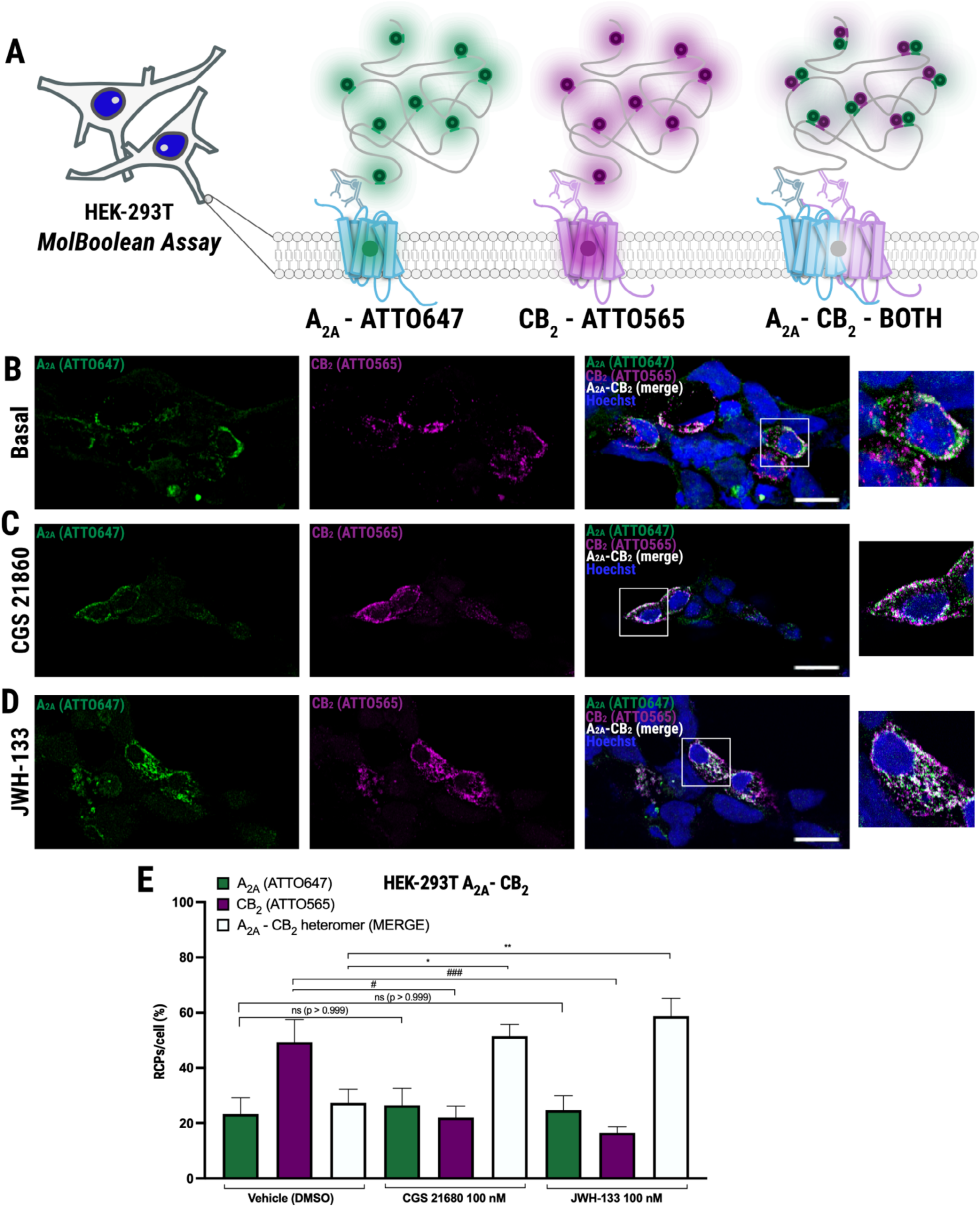
MolBoolean analysis of A_2A_–CB_2_ heteromer redistribution in HEK‐293T cells upon agonist treatment. (A) Schematic representation of the MolBoolean assay for detecting A_2A_^free^ (green), CB_2_^free^ (magenta), and A_2A_–CB_2_^het^ (white). Representative confocal microscopy images of cells treated for 1 h with DMSO‐containing vehicle, used because, as indicated in Methods, the agonists stock solutions were prepared in DMSO (B), 100 nM CGS 21680 (C), or 100 nM JWH‐133 (D). Green: A_2A_R (ATTO647); Magenta: CB_2_R (ATTO565); Blue: Nuclei (Hoechst 33342). Scale bar = 20 μm. Insets show zoomed regions. (E) Quantification of the relative percentage of RCPs per cell corresponding to A_2A_^free^, CB_2_^free^, and A_2A_–CB_2_^het^. Data represent mean ± SEM (*n* = 3 independent experiments with multiple‐cell imaging). Statistical significance was determined by one‐way ANOVA followed by Dunnett's post hoc test (*F*(8, 42) = 7.59; global *p* < 0.001). ns: Non‐significant (*p* > 0.999) vs. *free* A_2A_R in vehicle (0.1% DMSO); #*p* = 0.039, ###*p* = 0.001 vs. CB_2_^free^ in vehicle (0.1% DMSO); *p = 0.03, **p = 0.006 vs. heteromer in vehicle (0.1% DMSO).

We next applied the MolBoolean assay in the same heterologous system to simultaneously quantify, at the single‐cell level, the relative proportions of rolling‐circle amplification products (RCPs) corresponding to *free* A_2A_R (ATTO647), *free* CB_2_R (ATTO565), and A_2A_–CB_2_ heteromers (both) (Figure 2A). For operational purposes (i) the fraction of adenosine receptors that are not detected with CB_2_ receptors will be denoted as A_2A_^free^, (ii) the fraction of cannabinoid receptors that are not detected with A_2A_ receptors will be denoted as CB_2_^free^, and (iii) the MolBoolean‐detectable A_2A_–CB_2_ heteromer‐associated signal will be denoted as A_2A_–CB_2_^het^.

The respective signal was abolished when omitting primary antibodies or probes (Supplementary Figure S2). In vehicle‐treated cells, all three populations were detectable, demonstrating the baseline coexistence of non‐interacting and interacting receptors (Figure 2B). Following 1 h agonist exposure with either CGS 21680 (Figure 2C) or JWH‐133 (Figure 2D), MolBoolean detected a significant shift in the distribution of total RCPs per cell. This shift was characterized by a marked increase in the proportion of A_2A_–CB_2_ receptor complexes, which mirrored a depletion in the pool of CB_2_^free^ signal fraction; interestingly, the decrease was promoted by either agonist (Figure 2E). To ensure accurate quantification of receptor partitioning in the heterologous system, only cells showing high expression of RCPs for both A_2A_ and CB_2_ receptors were included in the analysis, thereby excluding cells that incorporated only one of the two cDNA plasmids during transfection.

The MolBoolean assay confirmed the presence of A_2A_–CB_2_ receptor complexes detected by PLA (Figure 1) and allowed simultaneous visualization of heteromeric and non‐heteromeric receptor populations. Following agonist treatment, the number of A_2A_–CB_2_ heteromer‐positive signals increased, whereas the population of non‐heteromeric CB_2_R decreased. These results indicate that receptor activation is associated with a shift in receptor distribution toward the heteromeric state.

### 2.3 Pro‐Inflammatory Microglial Activation Promotes a High Level of A_2A_ –CB_2_ Heteromer Formation, Rendering Further Complex Formation Largely Refractory to Agonist Modulation

**FIGURE 3:**
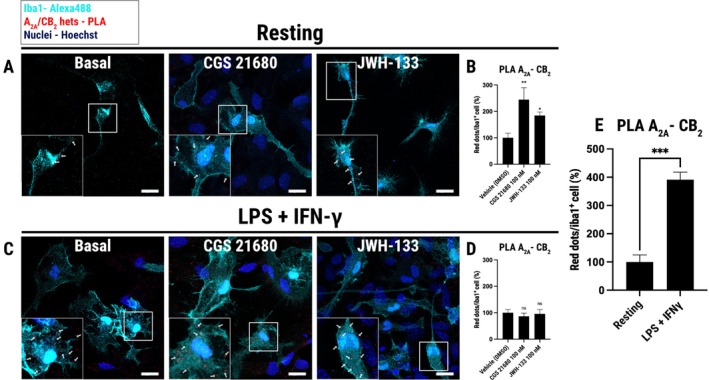
Activation upregulates A_2A_–CB_2_ heteromerization and alters agonist‐promoted heteromerization in microglial cells. Proximity Ligation Assays (PLA) were performed in primary microglial cells identified by Iba1 staining (cyan, Alexa 488). Representative confocal microscopy images of resting microglia (A) and of microglia treated with LPS (100 ng/mL) + IFN‐γ (20 ng/mL) for 48 h (C). Both resting and activated microglia were treated (1 h) with vehicle or agonists: 100 nM CGS 21680 or 100 nM JWH‐133. Red dots indicate A_2A_–CB_2_ heteromers; nuclei are stained with Hoechst 33342 (blue). Insets show zoomed regions with arrows highlighting PLA signals. Scale bar = 20 μm. Quantification of PLA signals, expressed as percentages relative to the respective vehicle condition (0.1% DMSO), in resting microglia (B). Data are presented as mean ± SEM from *n* = 3 independent experiments, with multiple cells analyzed per replicate. Statistical significance was assessed by one‐way ANOVA followed by Dunnett's post hoc test. This analysis revealed a significant overall treatment effect (*F*(2,25) = 6.84; global *p* = 0.004). Post hoc comparisons showed significant differences versus vehicle for 100 nM CGS 21680 **p = 0.002 and 100 nM JWH‐133 *p = 0.049 treatments. Quantification of PLA signals, expressed as percentages relative to the respective vehicle condition (0.1% DMSO), in activated microglia (D). Overall, the efffect of treatments did not reach statistical significance (*F*(2,26) = 0.27; global *p* = 0.768). Post hoc comparisons vs. vehicle were also non‐significant for CGS 21680 (*p* = 0.702) and JWH‐133 (*p* = 0.965) treatments. E: Comparison of basal A_2A_−CB_2_ heteromer levels (red dots/Iba1^+^ cell) in untreated resting or activated cells (normalized to resting). Statistical significance was determined by an unpaired, two‐tailed Student's *t*‐test with Welch's correction (*t* (4,6) = 7.99; ***p < 0.001 vs. resting). Data are presented as mean ± SEM from *n* = 3 independent experiments, with multiple cells analyzed per replicate.

We next examined the presence and distribution of endogenous A_2A_–CB_2_ heteromers in primary microglia. We induced the pro‐inflammatory phenotype by a 48 h treatment with LPS (100 ng/mL) and IFN‐γ (20 ng/mL); cells adopted a classical activated morphology, shifting from a ramified to an amoeboid shape, with significant increases in cell solidity and soma area, as assessed by Iba1 staining (Supplementary Figure S3). In resting microglia, PLA revealed discrete puncta, confirming that endogenous A_2A_R and CB_2_R form detectable complexes under basal conditions (Figure 3A). Incubation of resting microglia for 1 h with either CGS 21680 or JWH‐133 significantly increased the density of PLA puncta (Figure 3A,B), demonstrating that agonists promote complex formation or stabilization under basal conditions. Strikingly, in activated cells, agonist modulation was strongly constrained: treatment with CGS 21680 or JWH‐133 failed to further elevate A_2A_–CB_2_ heteromer density, suggesting that pro‐inflammatory activation shifts the detectable receptor‐associated signal toward a highly heteromerized basal configuration (Figure 3C,D). This plateau‐like response indicates that, in activated microglia, the dynamic range for further agonist‐induced increases in detectable A_2A_–CB_2_ complexes is markedly reduced. In fact, pro‐inflammatory activation alone triggered a marked rise (~4‐fold) of PLA signals relative to resting cells (Figure 3E), indicating that inflammation is a potent driver of heteromer formation or stability. Nevertheless, because PLA detects only interacting receptor pairs, the basis for the lack of agonist effect in activated cells remained unresolved. This issue was therefore addressed using MolBoolean analysis.

### 2.4 Activation Biases Microglial A_2A_ and CB_2_ Receptors Toward Heteromer Fractions

**FIGURE 4:**
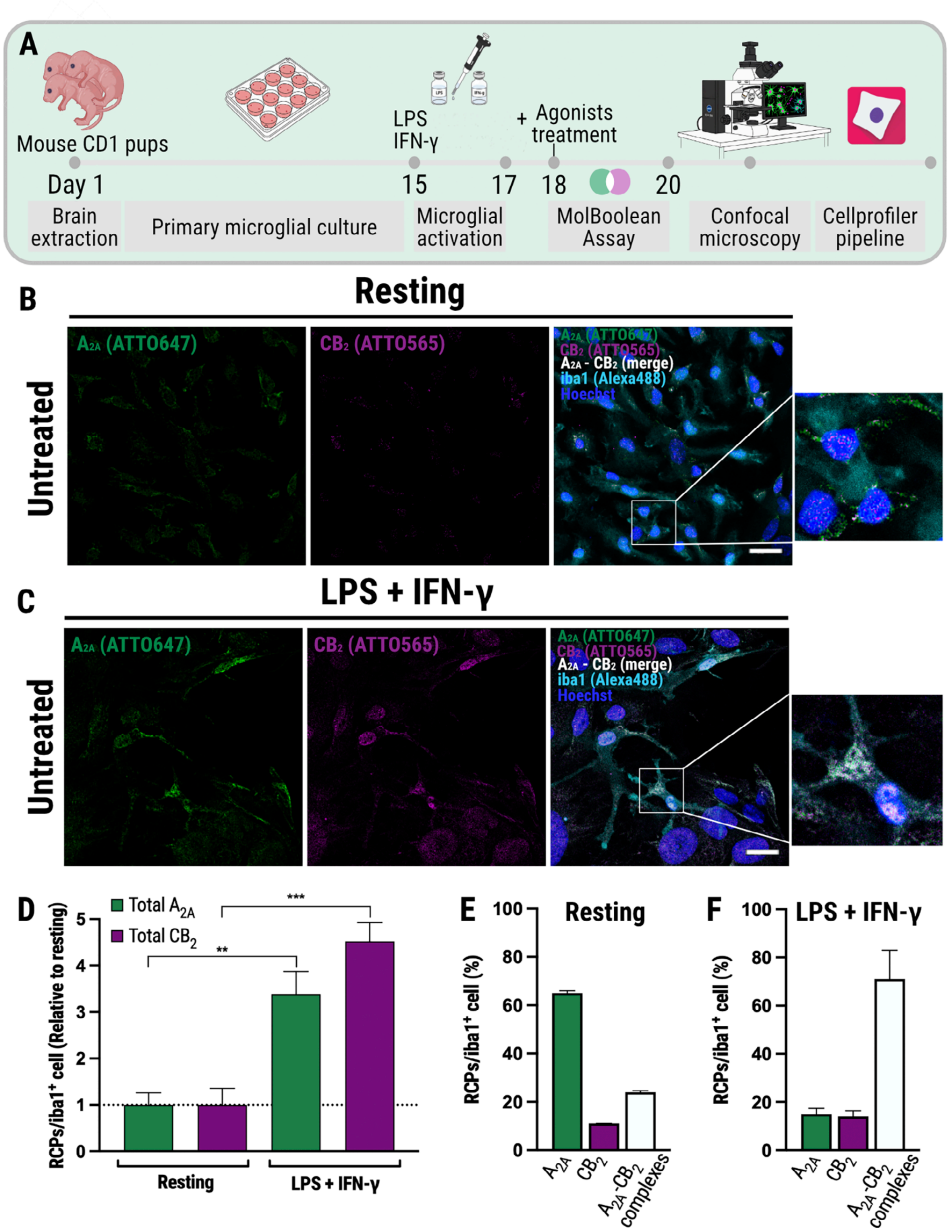
Pro‐inflammatory activation increases MolBoolean‐detectable A_2A_‐ and CB_2_‐receptor‐associated signal and shifts receptor partitioning toward the A_2A_–CB_2_^het^ fraction in microglia. MolBoolean assays were performed on resting microglia or on microglia activated for 48 h with LPS + IFN‐γ. (A) Experimental timeline scheme of primary cell preparation, activation, and MolBoolean assay procedure. Representative confocal microscopy images of MolBoolean assays in resting (B) and activated (C) cells. Confocal images show the signal for A_2A_R (ATTO647, green), CB_2_R (ATTO565, magenta), A_2A_–CB_2_ heteromers (merge, white) and with the microglial marker Iba1 (Alexa Fluor 488, cyan) and nuclei (Hoechst, blue). Insets show zoomed cells. Scale bar = 20 μm. (D) Quantification of the total number of RCPs per cell for A_2A_ and CB_2_ receptors (non‐interacting + interacting pools). Data are expressed as fold change relative to the signal quantified in resting cells and are presented as mean ± SEM from *n* = 3 independent experiments. Statistical significance was assessed by one‐way ANOVA followed by Tukey's post hoc test, revealing a significant overall effect (*F*(3,9) = 20.72; global *p* < 0.001). **p < 0.005 and ***p < 0.001 vs. resting cells. Quantification of the relative distribution (%) of RCPs corresponding to A_2A_^free^, CB_2_^free^ and A_2A_–CB_2_^het^ in resting (E) and activated (F) cells. Bars represent the mean percentage ± SEM of total RCPs per Iba1^+^ cell. Data shown in each graph were obtained from multiple cell images across *n* = 3 independent experiments.

To disentangle expression‐like effects, defined here as changes in total MolBoolean‐detectable receptor‐associated RCP signal, from partitioning effects, defined as changes in the distribution of that signal between non‐interacting and heteromer‐associated states, we employed the MolBoolean assay in primary microglia, following the experimental timeline outlined in Figure 4A. Total MolBoolean‐detectable receptor‐associated signal was estimated by summing, for each receptor, the RCPs assigned to its non‐interacting pool and the RCPs assigned to the A_2A_–CB_2_ heteromer‐associated pool. Thus, the total A_2A_R‐associated signal was calculated as A_2A_^free^ RCPs plus A_2A_–CB_2_^het^‐associated RCPs, whereas the total CB_2_R‐associated signal was calculated as CB_2_^free^ RCPs plus A_2A_–CB_2_^het^‐associated RCPs.

This analysis revealed a distinct landscape compared to that in transfected HEK‐293T cells (Figure 2). In resting microglia, MolBoolean analysis revealed that the A_2A_R‐associated signal markedly exceeded the CB_2_R‐associated signal. Although this does not establish receptor stoichiometry, it is consistent with a larger pool of A_2A_R‐associated signal and a heteromer fraction potentially limited by the lower detectable CB_2_R‐associated signal (Figure 4B,E).

Pro‐inflammatory activation with LPS and IFN‐γ (Figure 4C) induced a dual response: (i) a significant increase in the total detectable levels of both receptors (Figure 4D), and (ii) an increased proportion of receptors associated with heteromeric complexes (Figure 4F). This distinction is particularly relevant because interaction‐only assays cannot discriminate between changes in total receptor abundance and changes in the fraction of receptors engaged in heteromers. Total receptor expression was estimated by summing *free* receptor‐associated RCPs, detected in green or magenta, with A_2A_–CB_2_^het^‐associated RCPs, detected in white, for each receptor in individual Iba1^+^ cells. Notably, the increase in CB_2_R‐associated signal was accompanied by a marked redistribution of detectable receptor‐associated signal toward the A_2A_–CB_2_^het^ fraction (Figure 4F). These results suggest that inflammatory conditions not only increase detectable receptor‐associated signal but also favor repartitioning toward heteromer‐associated assemblies. This dual effect was particularly evident for CB_2_R. Although activation increased the total MolBoolean‐detectable CB_2_R‐associated signal by more than 4‐fold (Figure 4D, purple bars), the relative proportion of non‐interacting CB_2_R‐associated signal remained low (~15%; Figure 4E,F). Thus, the activation‐induced increase in detectable CB_2_R‐associated signal was not accompanied by a proportional expansion of CB_2_^free^. Instead, a larger fraction of the detectable CB_2_R‐associated signal was represented within A_2A_–CB_2_^het^ signal. In activated microglia, approximately 70% of the detectable receptor‐associated signal corresponded to A_2A_–CB_2_ heteromers.

### 2.5 Agonist‐Driven A_2A_ and CB_2_ Receptor Repartitioning is Constrained in Activated Microglia

**FIGURE 5:**
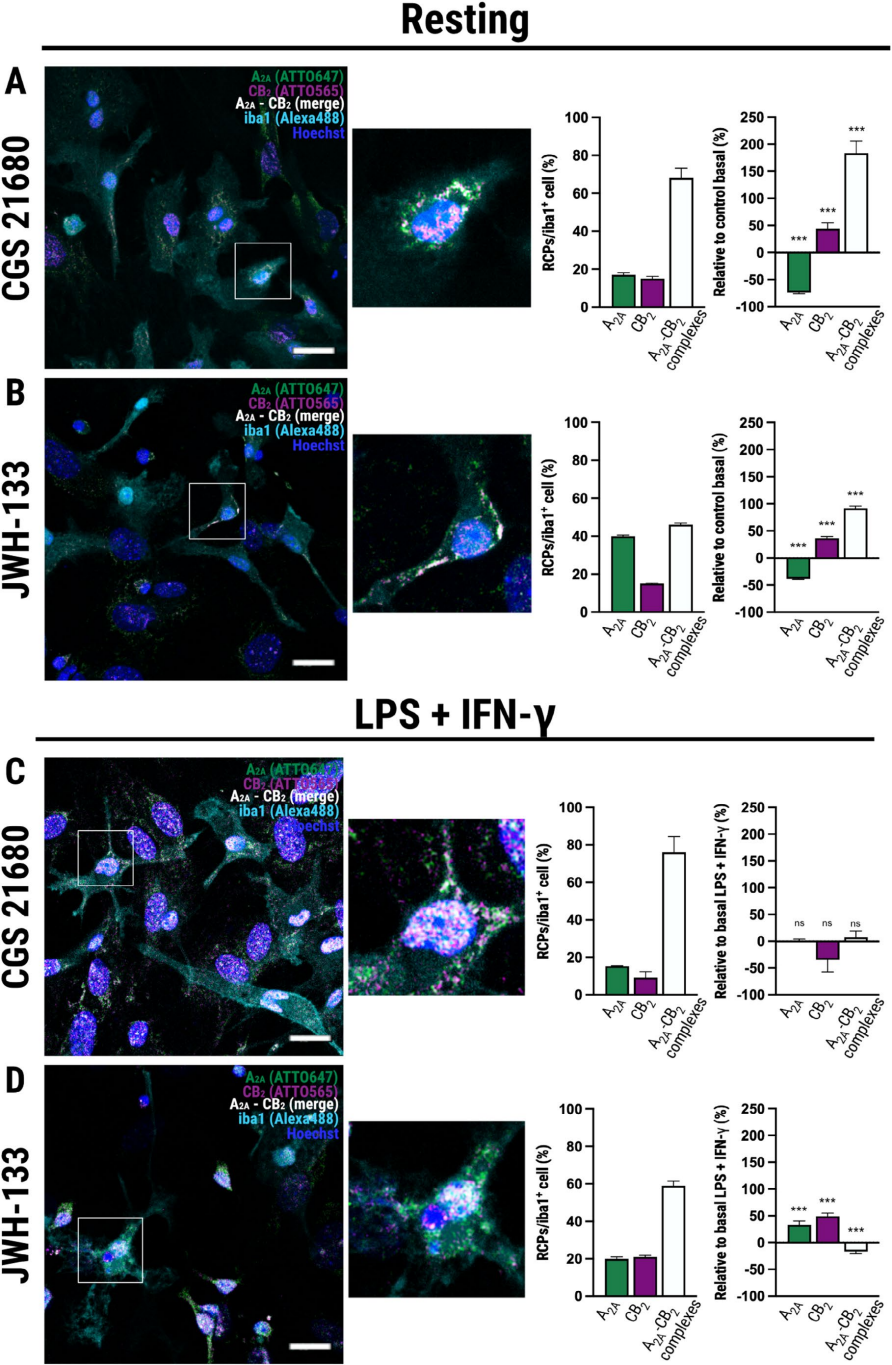
Effect of selective agonists on receptor‐associated signal partitioning in resting and activated microglia. MolBoolean assays were performed on resting (Control, A, B) and activated microglia (C, D) treated for 1 h with 100 nM CGS 21680 (A, C) or 100 nM JWH‐133 (B, D). Representative confocal microscopy images and corresponding quantifications of RCP distribution (%) in resting microglia treated with CGS 21680 (A) or JWH‐133 (B). Graphs on the right show the relative change (%) of each RCP population compared to untreated resting cells (data shown in Figure 4). Insets show zoomed cells. Scale bar = 20 μm. Representative confocal microscopy images and corresponding quantifications of RCP distribution (%) in LPS + IFN‐γ activated microglia treated with CGS 21680 (C) or JWH‐133 (D). Graphs on the right show the relative change (%) of each RCP population compared to untreated activated cells (data shown in Figure 4). Bars represent mean ± SEM from multiple cell images across *n* = 3 independent experiments. Statistical significance of the relative changes (%) versus untreated baseline (0) was determined by a one‐sample *t*‐test. For resting microglia treated with either agonist (A, B) and activated microglia treated with JWH‐133 (D), receptor redistributions were highly significant (***p < 0.001 vs. baseline). In contrast, in activated microglia, CGS 21680 failed to induce significant repartitioning (ns: Non‐significant, *p* > 0.05 vs. basal).

We next asked whether selective activation of A_2A_ or CB_2_ receptors differentially reshapes receptor partitioning between non‐interacting and heteromer‐associated receptor signal fractions in resting versus pro‐inflammatory microglia. In resting cells (Figure 5A,B), treatment with 100 nM CGS 21680 or 100 nM JWH‐133 redistributed signal among the three detectable populations, with a pattern distinct from that observed in transfected HEK‐293T cells. To quantify this redistribution, we analyzed the relative change of each population compared to the resting basal condition (Figure 4E). Specifically, CGS 21680 markedly decreased the A_2A_^free^ fraction, resulting in an increase in the fraction of A_2A_–CB_2_ receptor complexes (Figure 5A). JWH‐133 produced an intermediate redistribution between untreated and CGS 21680‐treated conditions (Figure 5B). Notably, the CB_2_^free^ fraction remained low across all resting conditions, and both agonists exerted broadly similar effects on its relative abundance.

**FIGURE 6:**
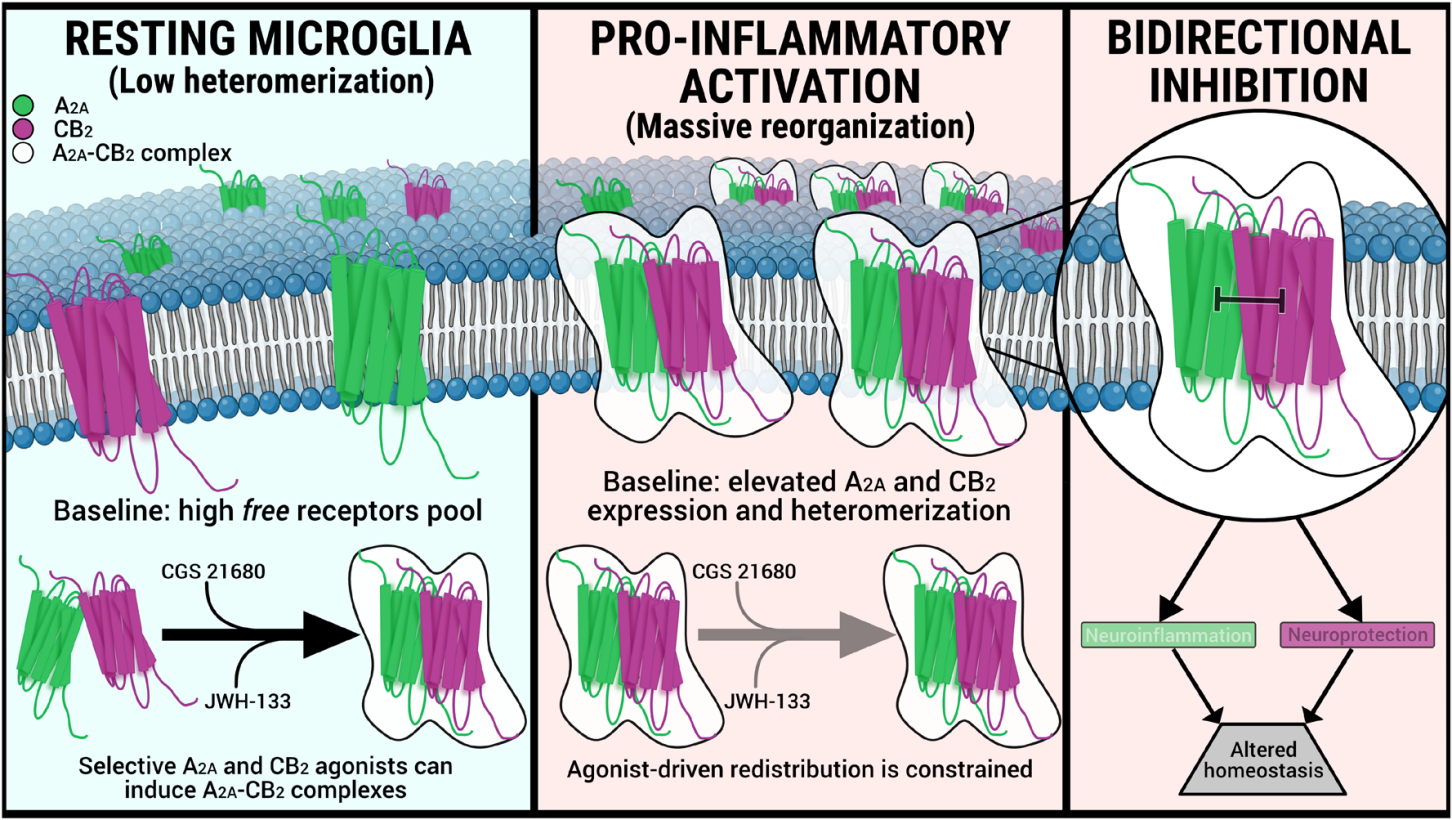
Proposed model of inflammation‐dependent repartitioning of A_2A_ and CB_2_ receptors. The schematic summarizes how pro‐inflammatory activation with LPS + IFN‐γ changes the organization of A_2A_ and CB_2_ receptors from a resting condition characterized by a substantial non‐interacting A_2A_ receptor population toward an activated condition in which a larger proportion of MolBoolean‐detectable receptor‐associated signal corresponds to A_2A_–CB_2_ heteromeric complexes. This model illustrates receptor repartitioning as a structural and organizational change in microglia. Potential consequences for A_2A_ and CB_2_ receptor signaling are inferred from previous functional studies and remain to be directly tested in the experimental paradigm used here.

In contrast, in LPS + IFN‐γ‐activated microglia, which are strongly enriched in the A_2A_–CB_2_^het^ signal fraction (Figure 4F), agonist effects were constrained (Figure 5C,D). Although CB_2_R activation with JWH‐133 still induced a statistically significant redistribution in activated microglia (Figure 5D), the magnitude of this effect was markedly reduced compared with resting conditions. These findings suggest that pro‐inflammatory activation constrains the capacity of the system to undergo further agonist‐induced redistribution, possibly because a large fraction of the detectable signal is already assigned to the A_2A_–CB_2_^het^ class. These data support a context‐dependent model in which pro‐inflammatory activation establishes a new baseline, characterized by elevated MolBoolean‐detectable A_2A_‐ and CB_2_‐associated signal and strong heteromer engagement, thereby limiting the available dynamic range for further agonist‐induced repartitioning. Importantly, neither agonist reduced A_2A_–CB_2_ heteromerization under any condition tested (Figure 6).

## 3 Discussion

The idea that class A GPCRs can physically interact to form higher‐order complexes was introduced more than four decades ago by Fuxe and Agnati, who proposed receptor–receptor interactions as an integrative mechanism in synaptic signaling (Agnati et al. 1982; Fuxe et al. 1983; Fuxe and Agnati 1985). Subsequent experimental studies established that class A GPCR heteromers can be detected in heterologous expression systems, primary cultures and native tissues, including brain specimens (Hébert and Bouvier 1998; Jordan and Devi 1999; Franco et al. 2000; Rocheville et al. 2000; Gines et al. 2000; Hillion et al. 2002; Lee et al. 2004; Borroto‐Escuela et al. 2014; George et al. 2014; Navarro et al. 2016; Rivas‐Santisteban et al. 2023). Together, these studies have established receptor heteromerization as a widespread organizational principle in GPCR biology.

Proximity ligation assay (PLA) has been instrumental in confirming the presence of GPCR heteromers in native cellular contexts and tissue samples (Gomes et al. 2016; Faron‐Górecka et al. 2019). However, conventional PLA reports only on receptor pairs located within molecular proximity and does not provide information on the proportion of receptors that remain outside the heteromeric complex. This limitation is important because, without quantitative information on how receptors are partitioned, interpretation of signaling integration, ligand responsiveness, and pharmacological targeting remains incomplete.

The present study addresses this fundamental gap by applying MolBoolean, a quantitative in situ method that allows simultaneous detection of non‐interacting receptors and heteromeric complexes at single‐cell resolution (Raykova et al. 2022). By resolving receptor partitioning, MolBoolean moves the GPCR heteromer field beyond the simple detection of receptor–receptor proximity and toward the quantitative analysis of receptor organization. Using this strategy, we show that A_2A_ and CB_2_ receptors coexist as mixed populations of A_2A_^free^, CB_2_^free^, and A_2A_–CB_2_^het^ in heterologous cells and in primary microglia. Importantly, this balance is not static, but is dynamically regulated by receptor activation and by inflammatory context. In resting microglia, *free* receptors predominate and A_2A_–CB_2_ heteromers represent a minority population; under these conditions, selective agonists promote receptor redistribution toward the heteromeric pool. By contrast, pro‐inflammatory activation with LPS and IFN‐γ induces receptor upregulation together with a marked repartitioning of detectable A_2A_ and CB_2_ receptor‐associated signals, such that approximately 70% become engaged in A_2A_–CB_2_ heteromers. Under these conditions, the capacity for further agonist‐induced redistribution is strongly constrained, reducing the dynamic range for ligand‐driven modulation.

These findings provide a quantitative framework for interpreting previous evidence that A_2A_–CB_2_ heteromers behave as integrated allosteric units rather than as simple physical aggregates. Functional and computational studies have shown that the A_2A_–CB_2_ heteromer imposes reciprocal constraints on both protomers. On the CB_2_ receptor side, heteromerization restricts transmembrane helix 6 (TM6) mobility required for canonical G_i_‐protein coupling, thereby attenuating cannabinoid receptor efficacy even in the presence of selective agonists (Llinas del Torrent et al. 2025). Conversely, A_2A_R‐mediated G_s_ coupling and downstream cAMP accumulation are negatively modulated by CB_2_R co‐activation within the heteromeric complex (Franco et al. 2019). Thus, both receptors acquire pharmacological and signaling properties within the heteromer that are qualitatively distinct from those of their corresponding non‐interacting fractions. Our data now anchor this allosteric model to a quantitative cellular context, showing that pro‐inflammatory activation can shift the receptor population toward a state in which heteromer‐associated signaling is expected to predominate.

Despite the extensive experimental evidence supporting GPCR heteromerization, its impact on drug discovery has remained limited, in part because high‐resolution three‐dimensional structures of class A GPCR heteromers remain scarce. *In silico* approaches based on solved GPCR structures have provided valuable predictions of transmembrane interaction interfaces, as exemplified by tools such as DIMERBOW (available at http://lmc.uab.es/dimerbow/; accessed on December 26, 2025) (García‐Recio et al. 2020), but experimental determination of GPCR heteromer structures remains highly demanding. Recent cryo‐electron microscopy studies of class C GPCRs have provided an important conceptual advance by showing how dimeric receptors can combine large extracellular‐domain rearrangements with direct transmembrane‐domain contacts. In the GABAB receptor, the obligatory GB1–GB2 heterodimer is largely organized by prominent extracellular Venus flytrap domains, whose agonist‐induced closure is transmitted through stalk regions to the transmembrane domains. Full‐length GABAB receptor structures revealed that activation involves rearrangement of the transmembrane dimer interface, including formation of a TM6–TM6 contact in the active state and stabilization by positive allosteric modulators bound at the transmembrane dimer interface (Mao et al. 2020; Shaye et al. 2020). Thus, although class C receptor dimerization seems to be mainly facilitated by large N‐terminal extracellular domains, these studies demonstrate that transmembrane interfaces are central to class C GPCR activation.

Class A GPCR heteromers are conceptually and technically distinct from obligate class C dimers. Unlike class C receptors, class A GPCRs generally lack large extracellular domains that impose and stabilize a predefined dimeric architecture. Their putative heteromeric interfaces are expected to rely predominantly on smaller, more dynamic transmembrane‐helix contacts within the lipid bilayer. Therefore, while the structural resolution of class C GPCR dimers provides hope that class A GPCR heteromers may eventually be solved, this task is likely to be considerably more difficult. The design of fusion, tandem or polycistronic constructs that faithfully reproduce endogenous stoichiometry, orientation and membrane organization remains particularly problematic for class A receptors. For this reason, proximity‐based in situ approaches such as PLA and MolBoolean remain indispensable for mapping class A GPCR heteromer organization in native cellular contexts.

The therapeutic targeting of A_2A_ and CB_2_ receptors in neurodegenerative and neuroinflammatory diseases has attracted considerable interest over the past two decades (Saura et al. 2005; de Lago and Fernández‐Ruiz 2007; Merighi et al. 2012; Wu et al. 2013; Madeira et al. 2015; Franco et al. 2019; Young and Denovan‐Wright 2022). A_2A_R antagonists, including istradefylline, have shown clinical efficacy in parkinsonian motor complications (Jenner et al. 2009; Kondo et al. 2015; Sako et al. 2017; Berger et al. 2020; Mori et al. 2022), whereas CB_2_R agonists have been widely explored as potential anti‐inflammatory or neuroprotective agents, with context‐dependent outcomes (Dhopeshwarkar and Mackie 2014; Soethoudt et al. 2017). Our findings suggest that microglial activation may alter the receptor context in which such ligands act because there is a shift of A_2A_ and CB_2_ receptors toward incorporation into A_2A_–CB_2_ heteromers. This repartitioning in proinflammatory contexts may help explain why the efficacy of A_2A_R‐ or CB_2_R‐targeting compounds can vary across experimental models and disease states. In this sense, receptor organization, and not only receptor expression, should be considered when interpreting pharmacological responses in neuroinflammatory settings.

These observations also have implications for the development of heteromer‐aware pharmacology. If pro‐inflammatory activation favors incorporation of A_2A_ and CB_2_ receptors into heteromeric complexes with distinct signaling properties, then the behaviour of ligands designed or selected solely on the basis of their activity at isolated protomers may not be fully predictable in activated microglia. This supports the view that GPCR heteromers can constitute specific pharmacological entities and that heteromer‐selective or context‐aware therapeutic strategies may be required to achieve greater molecular precision (Casadó et al. 2009; Orru et al. 2011; Faron‐Górecka et al. 2019; Dale et al. 2022; Franco and Navarro 2023). This principle may extend beyond the A_2A_–CB_2_ pair to other pharmacologically relevant A_2A_R‐containing complexes, including A_2A_–D_2_ and A_2A_–mGlu5 receptor heteromers in basal ganglia circuits, where receptor organization is also expected to shape drug responses (Ferré et al. 2002; Fuxe et al. 2003; Borroto‐Escuela et al. 2018).

A number of limitations and future directions should be acknowledged. First, although MolBoolean resolves receptor partitioning in situ, it does not independently determine ligand binding affinities, such as *K*_*D*_ or *K*_*i*_, for non‐interacting versus heteromer‐engaged receptor pools. Second, the present study does not directly prove the functional consequences of inflammation‐induced repartitioning; rather, it provides a quantitative organizational framework that should guide future functional and in vivo studies. Third, our proximity‐based imaging approach is specifically designed to distinguish non‐interacting from heteromer‐associated receptor pools at the single‐cell level, a feature that cannot be directly validated by bulk expression methods such as Western blotting or flow cytometry. Although the specificity of the assay is supported by prior antibody validation and omission controls, future genetic validation using CRISPR/Cas9 or siRNA approaches would further strengthen this framework. Finally, while the present study focuses on selective agonists, the ability of antagonists or inverse agonists to prevent heteromer formation, stabilize specific receptor conformations or dissolve pre‐existing inflammation‐induced complexes remains an important question for future precision pharmacology.

In conclusion, this study shows that microglial A_2A_ and CB_2_ receptor organization is dynamically regulated by both ligand activation and inflammatory state. By demonstrating that activation of primary microglia can shift receptor‐associated signals from predominantly non‐interacting receptors toward A_2A_–CB_2_ heteromers, our findings emphasize that receptor distribution is a critical determinant of GPCR pharmacology in resident innate immune cells of the central nervous system. More broadly, the work supports a transition in the GPCR heteromer field from detecting receptor proximity to quantifying receptor organization, an essential step for understanding and therapeutically exploiting heteromer‐dependent signaling in neuroinflammatory disease.

## 4 Material and Methods

### 4.1 Reagents

CGS 21680 (A_2A_R agonist, ref. C141), JWH‐133 (CB_2_R agonist, ref. SML3627), PolyEthylenImine (PEI, ref. 408727), lipopolysaccharides (ref. L2880), interferon‐γ (ref. I4777), and Hoechst 33342 (ref. 14533) were purchased from Merck (St Louis, MO, USA).

Concentrated (10 mM) stock solutions of receptor ligands were prepared in DMSO (Merck ref. D4540; St Louis, MO, USA) and were stored at −20°C; they were thawed and diluted before use. In vehicle‐treated samples, DMSO was used at the same concentration as in the agonist‐treated conditions.

### 4.2 Antibodies

The antibody pair was selected to optimize both receptor specificity and compatibility with the methodology used for detection. The primary antibodies used were rabbit anti‐A_2A_R AB1559 (Merck) and mouse monoclonal anti‐CB_2_R sc‐293188/3C7 (Santa Cruz Biotechnology). This rabbit/mouse antibody combination was selected because it is compatible with species‐specific PLA and MolBoolean probes. This strategy was chosen to maximize specificity, signal quality, and assay compatibility. The antibodies have been used in different laboratories for preclinical studies; recent papers are (Rivas‐Santisteban et al. 2025) and (Yu et al. 2025) for, respectively, AB1559 and sc‐293188/3C7 antibodies.

### 4.3 Cell Culture

HEK‐293T cells, batch 70,022,180, were acquired from the American Type Culture Collection (ATCC). Cells were amplified and frozen in liquid nitrogen in several aliquots.

Cells from each aliquot were used until passage 18. HEK‐293T cells were grown in Dulbecco's modified Eagle's medium (DMEM) (ref. 11995040) supplemented with 2 mM L‐glutamine (ref. 25030081), 100 μg/mL sodium pyruvate (ref. 11360070), 100 U/mL penicillin/streptomycin (ref. 15140122), MEM non‐essential amino acids solution (1:100) (ref. 11140050), and 5% (*v*/v) heat‐inactivated fetal bovine serum (FBS) (ref. A5256701) (all supplements were from Gibco, Paisley, Scotland, UK) and maintained at 37°C in a humid atmosphere of 5% CO_2_.

### 4.4 Cell Transfection

HEK‐293T cells were transiently transfected with the corresponding cDNA by the PEI (Polyethylenimine; 40,872–7; Merck) method. Cells were transiently cotransfected with a constant amount of cDNA encoding for the A_2A_R (1.5 μg) and/or the CB_2_R (1.5 μg). Briefly, cDNAs diluted in 150 mM NaCl were mixed with PEI (5.5 mM), also prepared in 150 mM NaCl, and then incubated for 10 min. The cDNA‐PEI complexes were transferred to HEK‐293T cells and were incubated for 4 h in a serum‐starved medium. Then, the medium was replaced by a fresh supplemented culture medium, and cells were maintained at 37°C in a humid atmosphere of 5% CO_2_. Forty‐eight hours after transfection, cells were washed and were treated as described in *Proximity ligation assay* or *MolBoolean assay* sections.

### 4.5 Isolation of Primary Microglia

Primary microglial cultures were prepared from the brain of 1‐day‐old (P1) CD‐1 mice, following previously described protocols (Franco et al. 2020; Rivas‐Santisteban et al. 2021). Briefly, brains were removed, and the meninges were carefully stripped away. The cortical tissue was dissected, minced, and dissociated by enzymatic digestion with 0.25% trypsin at 37°C for 30 min, followed by mechanical trituration. The resulting cell suspension was plated in 12‐well plates with glass coverslips in Dulbecco's modified Eagle's medium (DMEM) supplemented with 10% FBS, 100 U/mL penicillin, 100 μg/mL streptomycin, and 2 mM L‐glutamine. Cultures were maintained at 37°C in a humidified 5% CO_2_ atmosphere.

After 12–14 days in vitro, when the mixed glial cultures reached confluence, microglia were used to perform the experiments. Cells were activated by treating resting cells with lipopolysaccharides (LPS, 100 ng/mL) and interferon gamma (IFN‐ γ, 20 ng/mL) for 48 h.

The purity of the microglial cultures was assessed by immunocytochemical staining with the specific marker Iba1 (ref. EPR16588, Abcam), showing > 90% purity. All experimental procedures were conducted according to the approved protocols from Generalitat de Catalunya (10571) following the experimental European Union guidelines and regulations (2010(63/EU)). Under current regulations, no specific protocol approval is required when animal tissues are used for the isolation of primary cells.

### 4.6 Proximity Ligation Assay

A_2A_−CB_2_ receptor proximity in HEK‐293T cells or primary microglia was assessed using the Duolink PLA detection Kit (ref. DUO92008, Sigma‐Aldrich; St Louis, MO, USA) following the instructions of the supplier. Cells were grown on glass coverslips and were treated for 1 h with CGS 21680 (100 nM) or JWH‐133 (100 nM) and, immediately after, washed with PBS and fixed in 4% paraformaldehyde (ref. 100,496, Merck; St Louis, MO, USA) for 15 min.

Then, cells were washed with PBS containing glycine (20 mM) to quench the aldehyde groups and permeabilized with the same buffer containing Triton X‐100 0.05% (ref. 142314.1611, PanReac; Barcelona, Spain) for 15 min and successively washed with PBS. Then, samples were incubated for 1 h at 37°C with a blocking solution (ref. DUO82007, Sigma Aldrich; St Louis, MO, USA) in a pre‐heated humidity chamber. Samples were incubated overnight in antibody diluent containing a mixture of equal amounts of rabbit anti‐A_2A_R (AB1559, Merck; St Louis, MO, USA) (1:200) and mouse anti‐CB_2_R (sc‐293188, Santa Cruz; Dallas, Texas) antibodies (1:200); then cells were incubated with Duolink in situ Probe anti‐Mouse PLUS (ref. DUO82001, Sigma‐Aldrich; St Louis, MO, USA) and anti‐Rabbit MINUS (ref. DUO82005, Sigma‐Aldrich; St Louis, MO, USA) for 1 h at 37°C. After that, ligation and amplification were conducted as indicated by the supplier. In all cases, nuclei were stained with Hoechst 33342 (1:1000 from 1 mg/mL stock; Merck; St Louis, MO, USA) for 5 min. Samples were preserved at 4°C in the dark using Epredia Immu‐mount (ref. 10622689, ThermoFisher; Waltham, MA, USA) until imaging. Confocal images were acquired on a Zeiss LSM 880 confocal microscope (Zeiss, Jena, Germany) using a 63× oil‐immersion objective (N.A. 1.4) and 405 nm (for nuclei detection) and 561 nm (for red‐dot detection) laser lines. In the case of microglial cells, to ensure that heteromer quantification was restricted to this cell type, Iba1–Alexa 488 (ref. EPR16588, Abcam) labeling was used. For each image, five Z‐planes with a step size of 1 μm were acquired.

For the quantification of A_2A_‐CB_2_ heteromers in the obtained PLA confocal images, a custom‐designed image analysis pipeline for CellProfiler was used. The pipeline included the following processing modules: “ColorToGray” to split each RGB image into the blue (nuclei), red (PLA dots) channels and, in the case of microglia, green (iba1^+^ cells); “GaussianFilter” applied to the blue channel to smooth and correct the shape of the nuclei, facilitating their subsequent detection; and “IdentifyPrimaryObjects,” used to detect all nuclei in the image by setting the expected minimum and maximum diameters and applying Otsu's thresholding method. Next, “IdentifySecondaryObjects” segmented the image by defining an approximate region surrounding each nucleus. In the red channel, the “EnhanceOrSuppressFeatures” module (with the enhance speckles option) improved the detection of PLA dots. “IdentifyPrimaryObjects” was then used again to apply an appropriate threshold for detecting these red dots. To associate detected PLA dots with individual nuclei, the “RelateObjects” module was used. To quantify PLA dots only in cells positive for iba1 labeling, the “MaskImage” module was used. Quality control images for each analyzed field were generated using the “OverlayOutlines” and “SaveImages” modules. Finally, all quantitative data were exported to a “.csv” file using the “ExportToSpreadsheet” module.

### 4.7 MolBoolean Assay

The MolBoolean assay kit was obtained from Atlas Antibodies (Stockholm, Sweden) and the manufacturer's protocol was followed. The specificity of the MolBoolean method to distinguish interacting from non‐interacting proteins in crowded environments has been rigorously validated in previous studies (Rivas‐Santisteban et al. 2023; Raykova et al. 2022).

Transfected HEK‐293T cells or primary microglia were washed using PBS and fixed with ice‐cold 4% formalin solution (ref. 1,00,969,011; Merck) for 15 min. Samples were washed three times with PBS and permeabilized with TBS (ThermoFisher Scientific) containing 0.2% v/v Triton X‐100 (ref. 142314.1611; Panreac) for 15 min. After washing for 2 min with TBS, the samples were transferred to a humid chamber. Samples mounted on glass microscope slides (SuperFrostPlus; ref. 631–0108; VWR) were outlined with a hydrophobic barrier using an A‐PAP pen (Z672548; Merck). Blocking was done with the appropriate solution supplied in the kit for 1 h at 37°C. The samples were incubated with a mouse monoclonal anti‐CB_2_R primary antibody (1:200, sc‐293,188, Santa Cruz; Dallas, Texas) for 2 h at RT. Afterwards samples were incubated (overnight at 4°C) with a rabbit polyclonal anti‐A_2A_R primary antibody (1:200; AB1559, Merck; St Louis, MO, USA). Primary antibodies were diluted in blocking solution. The samples were then washed 3 times (3 min each) with TBS containing 0.05% Tween‐20 (TBST; ref. P5927; Merck) and incubated (for 1 h at 37°C) with 3 μg/mL of each proximity probe (A and B), diluted in intercept blocking solution. Next, samples were washed once during 3 min in HBS‐Tween‐20 (HBST) and twice during 3 min in TBST. Subsequently, the cells were incubated (1 h at 37°C) with 0.05 μM oligonucleotide sequence in T4 DNA ligase buffer supplemented with 0.25 mg/mL BSA (Merck), followed by a 3 min wash with HBST and a 3 min wash with TBST. Later, a mix of 0.125 U/μL Nt.BsmAI (nickase enzyme) in NEBuffer CutSmart (New England Biolabs) and 0.25 mg/mL BSA was added (30 min at 37°C). For the hybridization of the tag oligonucleotides, the samples were washed with TBST and then were incubated (1 h at 37°C) in TBS, 0.25 mg/mL BSA and 0.5 μΜ tag oligonucleotides A and B. Finally, ligation was achieved using 0.05 U/μL T4 ligase in T4 DNA ligase buffer containing 0.25 mg/mL BSA (1 h at 37°C). Washed samples were incubated (90 min at 37°C) in phi29 polymerase buffer (Monserate Biotechnology group; San Diego, CA), 0.25 mg/mL BSA, 1.25 mM dNTPs (Thermo Fisher Scientific), and 1 U/μL phi29 polymerase (Monserate Biotechnology group). Further washes with TBST preceded incubation (1 h at 37°C) with detection mix (0.025 μΜ detection oligonucleotides A and B dissolved in TBS). Excess reagent was removed using HBS and later, TBS. Nuclear counterstaining was conducted using Hoechst 33342 (1:1000; ThermoFisher) for 5 min.

Samples were preserved with Shandon Immu‐Mount (9,990,402; ThermoFisher) and were observed in a Zeiss 880 confocal microscope (Carl Zeiss, Oberkochen, Germany) equipped with an apochromatic 63× oil immersion objective (N.A. 1.4) and 405, 488, 561, and 640 nm laser lines. For each field of view, a stack of three or four channels (405 nm for nuclei in blue, 561 nm for the CB_2_R in magenta and 640 nm for the A_2A_R in green with or without 488 nm for iba1 microglial marker in cyan) and images from a minimum of 3 *Z* stacks with a step size of 0.5 μm were acquired and quantified independently.

### 4.8 Specificity Controls

The primary antibodies used in this study have previously been shown to be suitable for detecting A_2A_ or CB_2_ receptors by Western blotting, immunofluorescence, and proximity‐based assays in both heterologous expression systems and native tissues (Navarro et al. 2018; Franco et al. 2019; Rivas‐Santisteban et al. 2021, 2023). Furthermore, no detectable immunofluorescence signal was observed in untransfected HEK‐293T cells. For both PLA and MolBoolean assays, negative controls were systematically performed by omitting one or both primary antibodies or by omitting the proximity probes (Supplementary Figures S1 and S2). Omission controls yielded no detectable signal, indicating that the observed PLA/RCP signals seem not to be attributable to nonspecific probe interactions, endogenous nucleic acid templates, or autofluorescence. Antibody target specificity is supported by the consistency of the findings reported across published studies, although genetic validation remains an important future refinement. Importantly, the state‐dependent repartitioning of these signals upon activation is evidence of on‐target biological specificity, as random off‐target background would not exhibit such dynamic, structured reorganization.

### 4.9 Data Analysis

Quantification and colocalization analyses of the rolling circle amplification products (RCPs) were performed with specifically designed pipelines for the CellProfiler software on the deconvolved images. Images were deconvolved with Parallel Spectral Deconvolution v1.12 plugin for ImageJ software applying Tikhonov's algorithm, after calculating the appropriate Point Spread Function (PSF) by considering the refraction index of the mounting medium, numerical aperture of the objective, and wavelength of the channel for deconvolution.

Deconvolved split‐channel images in grayscale format were analyzed using the CellProfiler software version 5.2.8. For MolBoolean image analysis, a pipeline for RCPs quantification, with slight modifications between assays to adjust the size for nuclei detection or the estimated area of each cell, was compiled with the following modules: IdentifyPrimaryObjects, IdentifySecondaryObjects, EnhanceOrSuppressFeatures, GaussianFilter, IdentifyPrimaryObjects, ExpandOrShrinkObjects, IdentifySecondaryObjects, CombineObjects, MaskObjects, MeasureObjectIntensity, ClassifyObjects, FilterObjects, OverlayOutlines, SaveImages, RelateObjects, and ExportToSpreadsheet.

First, IdentifyPrimaryObjects was used on the Hoechst stain channel (blue; ch00) to identify nuclei based on their typical diameter measured in pixels and the application of two‐class Otsu thresholding. Next, the IdentifySecondaryObjects module was used to identify the cells, by means of expanding the nuclei by distance. GaussianFilter module was used with low sigma value to effectively reduce noise, preserving fine details but improving image quality for subsequent RCPs analysis on ATTO565 (magenta; ch01) and ATTO647 (green; ch02) channels. Then, the EnhanceOrSuppressFeatures module was used to improve specks (RCPs) and the IdentifyPrimaryObjects module to quantify RCPs of a specific diameter in pixels. The ExpandOrShrinkObjects module was used to shrink objects to a single point, while the IdentifySecondaryObjects module was used to optimize RCPs delimitation. With the new RCPs definition, the CombineObjects module merged all blobs in ch01 and ch02 channels and MaskObjects excluded from the analysis all the RCPs outside the previously defined cells. The intensity of the RCPs in ch01 and ch02 channels was determined with the MeasureObjectIntensity module and, with the ClassifyObjects module, identified RCPs were classified as background (low intensity for both channels), as CB_2_ RCPs (high intensity for ATTO565, low intensity for ATTO647), as A_2A_ RCPs (low intensity for ATTO565, high intensity for ATTO647), or as A_2A_‐CB_2_ heteromer RCPs (high intensity for both channels). RCPs were then filtered with the FilterObjects module, in A_2A_^free^, CB_2_^free^, and A_2A_–CB_2_^het^, with the option of saving this information in an image using OverlayOutlines and SaveImages modules. Afterwards, the RCPs were matched to their appropriate cell using the RelateObjects module. Finally, all data collected from the execution of the pipeline was exported to a “.csv” file using the ExportToSpreadsheet module.

Some modifications were necessary to the above‐described pipeline to facilitate MolBoolean RCPs quantification in microglial cells. The main purpose was to quantify RCPs exclusively in cells labeled with the iba1 microglial marker. To achieve this, the Threshold module was used to convert Alexa Fluor 488 channel (ch03) into a binary image and for automatic structure segmentation using the minimum cross‐entropy thresholding method. After that, the MaskImage module was used to generate new images for ch01 and ch02 channels containing only the RCPs enclosed in the mask generated with the Threshold module. Quality control of the segmentation was performed with the SaveImages module.

Brightness and contrast were only adjusted for figure images; all quantification was performed on the unadjusted images. Pseudo‐coloring was applied to images in Figures; Hoechst 33342, Alexa Fluor 488, ATTO565, and ATTO647, are depicted, respectively, in blue, cyan, magenta, and green; pseudocolors were used for display only.

### 4.10 Statistical Analysis

Data are expressed as mean ± standard error of the mean (SEM) from at least three independent experiments (*n* ≥ 3 independent microglial preparations or HEK‐293T transfected cells), with multiple cells analyzed per replicate. Normality was assessed using the Shapiro–Wilk test prior to parametric analysis. Pairwise comparisons were performed using an unpaired, two‐tailed Student's *t*‐test. For relative changes (%) in signal partitioning, statistical significance against baseline (0, assigned to untreated) was evaluated using a one‐sample *t*‐test. Multiple group comparisons were analyzed by one‐way ANOVA followed by Dunnett's post hoc test, using the untreated/vehicle condition as the reference group. The F‐statistic and corresponding degrees of freedom were determined for all ANOVA comparisons. Statistical analyses and data fitting were performed using GraphPad Prism version 10.6.1 (GraphPad Software, San Diego, CA, USA). Significance thresholds were set at *p < 0.05, **p < 0.01, and ***p < 0.001, while non‐significant differences (*p* > 0.05) were denoted as ‘ns’. Exact *p*‐values are reported in the respective figure legends.

## Author Contributions

Conceptualization: Rafael Rivas‐Santisteban and Rafael Franco. Supervision: Rafael Rivas‐Santisteban, Jaume Lillo, and Christian Griñán‐Ferré. Validation: Rafael Rivas‐Santisteban and Rafael Franco. Investigation (experiments): Jaume Lillo and Rafael Rivas‐Santisteban. Resources: Rafael Franco, Christian Griñán‐Ferré, and Mercè Pallàs. Data Analysis: Rafael Rivas‐Santisteban. Writing – original draft: Rafael Rivas‐Santisteban and Rafael Franco. Figures and visualization: Rafael Rivas‐Santisteban, Rafael Franco, and Mercè Pallàs. Writing – review and editing: Rafael Rivas‐Santisteban, Jaume Lillo, Rafael Franco, Christian Griñán‐Ferré, and Mercè Pallàs. All authors edited and approved the final version of the manuscript.

## Funding

This work was funded by the Agencia Estatal de Investigacion (grant Ref. No. PID2021‐126600OB‐I00).

## Conflicts of Interest

The authors declare no conflicts of interest.

## Supporting information


**Figure S1:** Controls of the Proximity Ligation Assays (PLA). Nuclei were stained with Hoechst 33342 (blue). In A‐B, HEK‐293T cells co‐transfected with plasmids encoding for A_2A_ and for CB_2_ receptors. The assay was performed omitting one primary antibody (A) or one of the PLA probes (B). In (C) images from PLA assay performed in non‐transfected HEK‐293T cells incubated with both primary antibodies and PLA probes. In all panels representative confocal microscopy images are shown.
**Figure S2:** MolBoolean assay specificity. MolBoolean assays were performed in HEK‐293T cells co‐expressing A_2A_ and CB_2_ receptors to assess the specificity of the primary antibodies and MolBoolean probes. Controls were performed by omitting primary anti‐A_2A_R (A) or anti‐CB_2_R (B) antibodies or omitting the MolBoolean A (C) or B (D) probes. The figure shows representative confocal microscopy images, and the quantification bar graphs (relative distribution of RCPs per cell). Green corresponds to A_2A_R (ATTO647), magenta to CB_2_R (ATTO565), and white to A_2A_R–CB_2_R heteromers (Merge). Nuclei were stained with Hoechst 33342 (blue). Scale bar = 20 μm. Bars represent mean ± SEM. Data represent analyses from multiple cell images across *n* = 3 independent experiments.
**Figure S3:** Morphological changes indicative of microglial activation. Primary microglial cells were treated for 48 h with vehicle (Control) or with LPS (100 ng/mL) plus IFN‐γ (20 ng/mL). Cells were then fixed and stained with Alexa fluor 488‐conjugated anti‐Iba1 antibody. (A) Schematic representation of the morphological parameters quantified. Cell solidity is calculated as the ratio of the cell area to its convex hull area; perimeter and soma area are also measured. Representative confocal microscopy images of Iba1‐stained microglia (cyan) in Control (B) and LPS + IFN‐γ (C) conditions. Insets show zoomed cells, and right panels display the corresponding processed outlines used for morphological analysis. Scale bar = 20 μm. Box and whisker plots show quantification of morphological parameters: cell solidity (D), perimeter (E), and soma area (F) for control versus activated microglia **p < 0.01, ***p < 0.001 (Unpaired *t*‐test). Data represent analysis from multiple cell images across *n* = 3 independent experiments.

## Data Availability

The data that support the findings of this study are available on request from the corresponding author. The data are not publicly available due to privacy or ethical restrictions.
