# Supplementary material for: Quantifying Heteromer Partitioning Reveals Inflammation‐Dependent Redistribution of Microglial Adenosine A2A and Cannabinoid CB2 Receptors
Source: Glia. 2026 Jul 21;74(9):e70200. doi: 10.1002/glia.70200 (PMC13389093; doi:10.1002/glia.70200)
Supplement: Supplementary file 1 — Figure S1: Controls of the Proximity Ligation Assays (PLA). Nuclei were stained with Hoechst 33342 (blue). In A‐B, HEK‐293T cells co‐transfected with plasmids encoding for A2A and for CB2 receptors. The assay was performed omitting one primary antibody (A) or one of the PLA probes (B). In (C) images from PLA assay performed in non‐transfected HEK‐293T cells incubated with both primary antibodies and PLA probes. In all panels representative confocal microscopy images are shown. Figure S2: MolBoolean assay specificity. MolBoolean assays were performed in HEK‐293T cells co‐expressing A2A and CB2 receptors to assess the specificity of the primary antibodies and MolBoolean probes. Controls were performed by omitting primary anti‐A2AR (A) or anti‐CB2R (B) antibodies or omitting the MolBoolean A (C) or B (D) probes. The figure shows representative confocal microscopy images, and the quantification bar graphs (relative distribution of RCPs per cell). Green corresponds to A2AR (ATTO647), magenta to CB2R (ATTO565), and white to A2AR–CB2R heteromers (Merge). Nuclei were stained with Hoechst 33342 (blue). Scale bar = 20 μm. Bars represent mean ± SEM. Data represent analyses from multiple cell images across n = 3 independent experiments. Figure S3: Morphological changes indicative of microglial activation. Primary microglial cells were treated for 48 h with vehicle (Control) or with LPS (100 ng/mL) plus IFN‐γ (20 ng/mL). Cells were then fixed and stained with Alexa fluor 488‐conjugated anti‐Iba1 antibody. (A) Schematic representation of the morphological parameters quantified. Cell solidity is calculated as the ratio of the cell area to its convex hull area; perimeter and soma area are also measured. Representative confocal microscopy images of Iba1‐stained microglia (cyan) in Control (B) and LPS + IFN‐γ (C) conditions. Insets show zoomed cells, and right panels display the corresponding processed outlines used for morphological analysis. Scale bar = 20 μm. Box and whisker plots [file GLIA-74-0-s001.docx]

**Quantifying heteromer partitioning reveals inflammation-dependent redistribution of microglial adenosine A_2A_ and cannabinoid CB_2_ receptors**

**Supplementary material**

Supplementary material provides standard controls for PLA (omission of one primary antibody or one probe; non-transfected cells) and for MolBoolean™ (omission of individual primary antibodies or probes), supporting signal specificity (Supplementary Figures S1 and S2). In addition, microglial activation induced the expected morphological changes, quantified using Iba1-based shape metrics (Supplementary Figure S3), validating the activated model and reinforcing the reliability of the MolBoolean/PLA-derived data and comparisons.


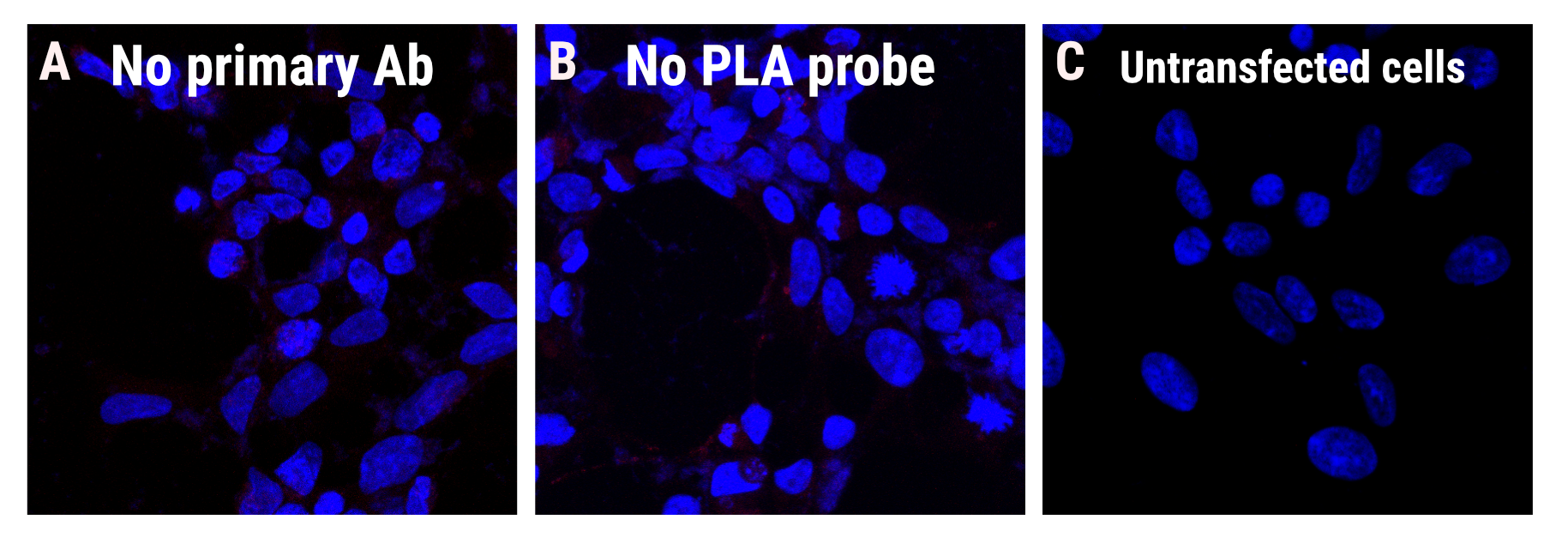


**Supplementary Figure S1. Controls of the Proximity Ligation Assays (PLA).** Nuclei were stained with Hoechst 33342 (blue). In A-B, HEK-293T cells co-transfected with plasmids encoding for A_2A_R and for CB_2_R. The assay was performed omitting one primary antibody **(A)** or one of the PLA probes **(B)**. In **(C)** images from PLA assay performed in non-transfected HEK-293T cells incubated with both primary antibodies and PLA probes. In all panels representative confocal microscopy images are shown.


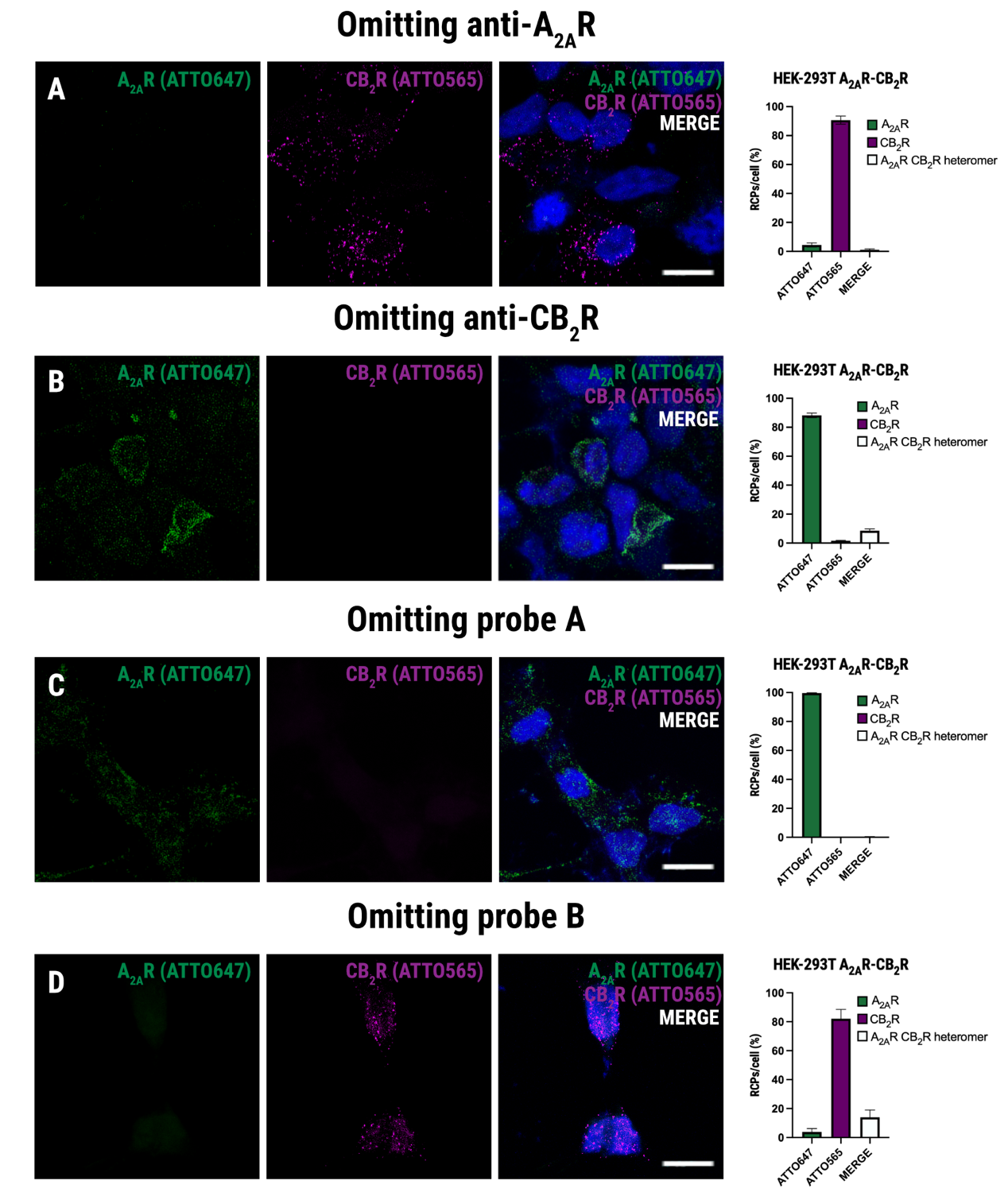


**Supplementary Figure S2. MolBoolean™ assay specificity.** MolBoolean™ assays were performed in HEK-293T cells co-expressing A_2A_ and CB_2_ receptors to assess the specificity of the primary antibodies, and of MolBoolean™ probes. Controls were performed by omitting primary anti-A_2A_R **(A)** or anti-CB_2_R **(B)** antibodies or omitting the MolBoolean™ A **(C)** or B **(D)** probes. The figure shows representative confocal microscopy images, and the quantification bar graphs (relative distribution of RCPs per cell), green corresponds to A_2A_R (ATTO647), magenta to CB_2_R (ATTO565), and white to A2AR–CB2R heteromers (Merge). Nuclei were stained with Hoechst 33342 (blue). Scale bar = 20 µm. Bars represent mean ± SEM. Data represents analysis from multiple cell images across n=3 independent experiments.


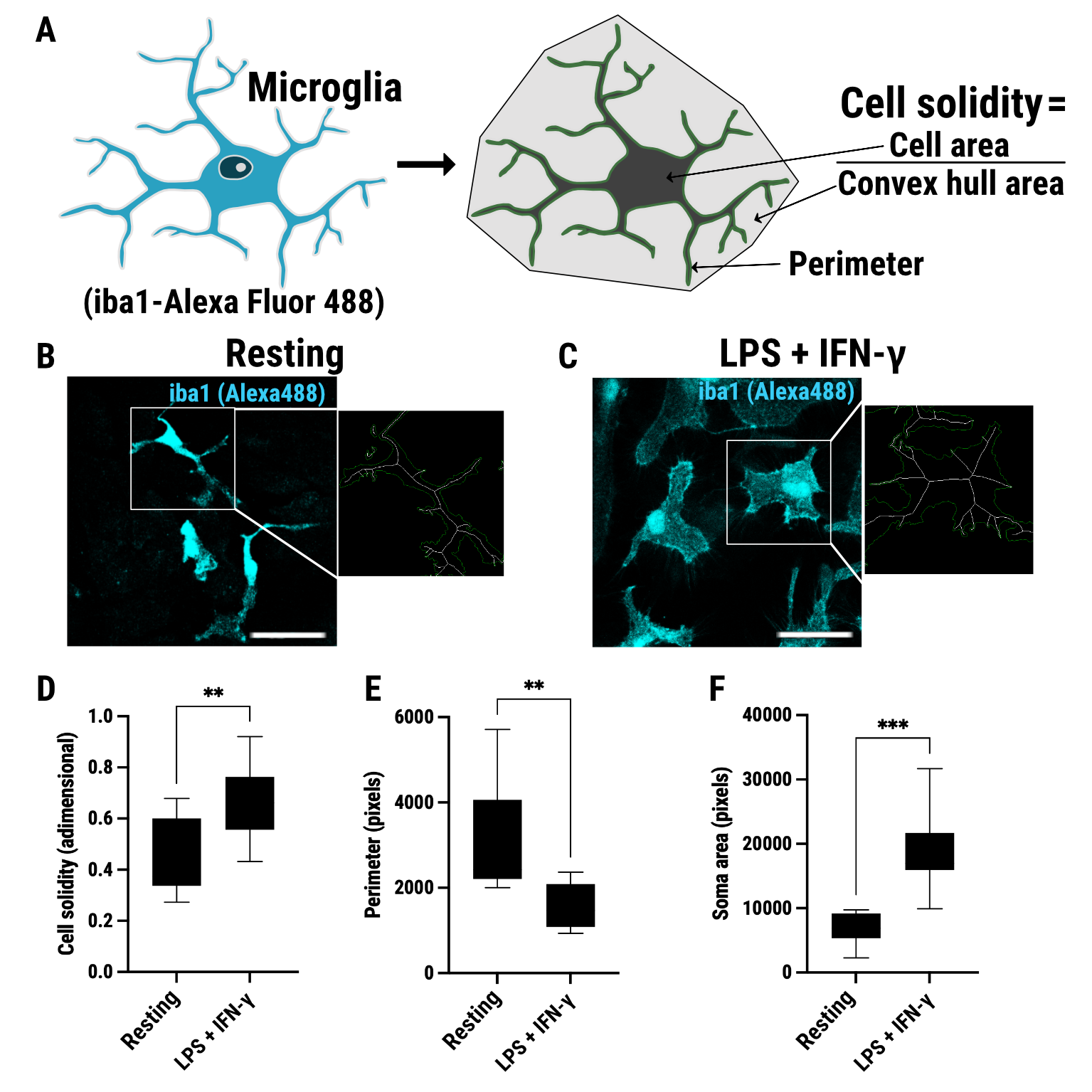


**Supplementary figure S3.** **Morphological changes indicative of microglial activation.** Primary microglial cells were treated for 48 hours with vehicle (Control) or with LPS (100 ng/mL) plus IFN-γ (20 ng/mL). Cells were then fixed and stained with Alexa fluor 488-conjugated anti-Iba1 antibody. **A)** Schematic representation of the morphological parameters quantified. Cell solidity is calculated as the ratio of the cell area to its convex hull area; perimeter and soma area are also measured. Representative confocal microscopy images of Iba1-stained microglia (cyan) in Control **(B)** and LPS + IFN-γ **(C)** conditions. Insets show zoomed cells, and right panels display the corresponding processed outlines used for morphological analysis. Scale bar = 20 µm. Box and whisker plots show quantification of morphological parameters: cell solidity **(D)**, perimeter **(E)**, and soma area **(F)** for control versus activated microglia **p<0.01, ***p<0.001 (Unpaired t-test). Data represent analysis from multiple cell images across n=3 independent experiments.
